# Restrictive surgical approach to palliate angina in a patient with coronary three vessel disease and biventricular metastatic hepatocellular carcinoma

**DOI:** 10.1186/s12957-017-1256-7

**Published:** 2017-12-07

**Authors:** Angela Kornberger, Tillmann Emrich, Andres Beiras-Fernandez, Christian-Friedrich Vahl

**Affiliations:** 1 Department of Cardiothoracic and Vascular Surgery, University Hospital, Johannes Gutenberg University, Langenbeckstr. 1, 55131 Mainz, Germany; 2 Department of Radiology, University Hospital, Johannes Gutenberg University, Mainz, Germany

**Keywords:** Metastatic cardiac tumor, Coronary artery disease, palliative surgery

## Abstract

**Background:**

Metastatic cardiac tumors may cause different symptoms including angina, symptoms of heart failure and/or arrhythmia. In patients with concomitant coronary artery disease, it may be difficult to distinguish between angina caused by metastases to the heart, for example, by stealing perfusion from the coronary arteries, and angina caused by coronary stenosis. Identifying the origin of the symptoms is, however, essential for designing appropriate surgical strategies.

**Case presentation:**

A 69-year-old male with multifocal recurrence of a hepatocellular carcinoma (HCC) presented with increasing ventricular arrhythmia and angina several weeks after posterior myocardial infarction and PCI of the RCA culprit lesion during which two further lesions present in the distal RCX and a posterolateral branch, and a chronically occluded LAD had not been addressed. MRI showed a large metastatic tumor infiltrating the walls of both ventricles as well as the interventricular septum. His debilitating symptoms were attributed to steal phenomena and/or perivascular compression caused by the metastatic tumor rather than the remaining coronary lesions, and he was offered a restrictive surgical approach consisting of debulking of the metastasis with an option for subsequent coronary intervention. The palliative surgical procedure resulted in a reduction of the tumor mass by half and sufficiently reduced the patient’s symptoms so that further coronary intervention was not required.

**Conclusions:**

Palliative surgery for metastases to the heart may benefit patients, provided that the origin of symptoms is identified correctly. It goes without saying that in a palliative setting, surgery should be limited to treating symptoms rather than performing extensive procedures addressing, for example, coronary artery or valve disease. Interventional cardiac procedures addressing not only CAD but also valve disease may supplement palliative tumor surgery.

## Background

Metastases of hepatocellular carcinoma (HCC) are most commonly found in the lung, bone and lymph nodes [1]. Cardiac metastases are rare. They often extend into the right atrium through the inferior vena cava [2] while isolated cardiac metastases are extremely rare. In this setting, curative surgical treatment is usually not possible, but resection or debulking of the cardiac tumor mass may be attempted to palliate symptoms resulting, for example, from steal phenomena due to perfusion being diverted from the coronary vessels into heavily vascularized tumor masses. In the case of simultaneous presence of severe coronary artery disease (CAD), however, it may be difficult to differentiate between angina caused by CAD and symptoms attributable the tumor, especially where a heavily vascularized tumor mass is present but no large tumor vessels communicating with the coronary arteries can be identified.

## Case presentation

A 69-year-old male who had undergone left-sided hemi-hepatectomy for HCC was diagnosed with multifocal hepatic recurrence of the tumor 1 year later. His cardiovascular risk profile consisted of smoking (38 pack years), arterial hypertension, insulin-dependent diabetes, obesity, and dyslipoproteinemia. His medical history additionally comprised bilateral carotid artery disease and stenting of the right internal carotid artery, chronic renal impairment, and chronic obstructive lung disease. Chronic occlusion and good collateralization of his left anterior descending artery (LAD) had been described 5 years previously when he underwent PCI for myocardial infarction with stent implantation in the RCX.

After recurrence of the HCC, he suffered a posterior NSTEMI and underwent stenting of a subtotal stenosis of the RCA. In addition to the chronically occluded LAD, coronary angiography now showed 80 and 90% stenosis of the distal RCX and a posterolateral branch, respectively, which were not addressed in the course of the intervention.

**Fig. 1:**
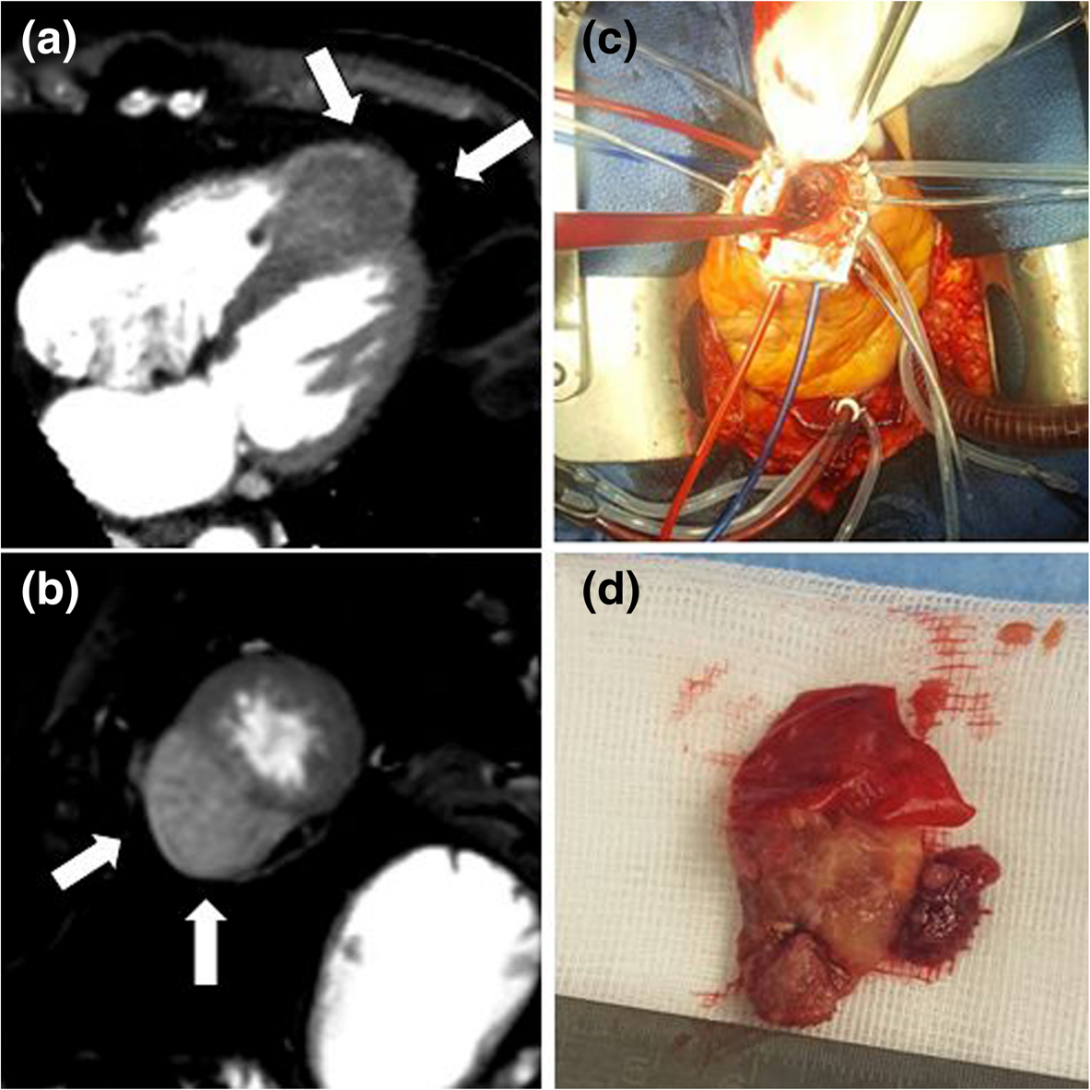
Preoperative imaging and intraoperative views of the metastatic tumor. **a** CT (256 Multislice Philips ICT, arterial phase, 4-chamber reconstruction images) and (**b**) MRI (Siemens Magnetom Prisma, 3 Tesla, T2 weighted single-shot TRUFI sequence) showing HCC metastasis infiltrating ventricular walls and septum, (**c**) intraoperative view showing apex with tumor exposed, (**d**) 27 × 27 × 12 mm section of tumor removed in toto

In the weeks after the coronary intervention, he suffered from increasing angina and developed ventricular arrhythmia. Echocardiography, MRI (Siemens Magnetom Prisma, 3 Tesla), and CT (256 Multisclice Philips ICT) showed a tissue mass of 72 × 45 × 56 mm infiltrating the myocardium of both ventricles and the interventricular septum (Fig. 1a, b). When his cardiac symptoms became so debilitating that they limited his quality of life more than the cancer and he expressed an urgent desire for treatment despite his dismal prognosis, he was referred to our department to evaluate surgical treatment options.

As the RCA lesion had already been addressed, the LAD had been occluded with good collateralization for at least 5 years, and we agreed with the cardiologist that it was highly unlikely that such severe symptoms were caused by the remaining RCX lesions alone, we assumed the metastatic tumor to contribute to the patient’s deterioration by stealing perfusion from, compressing, or infiltrating coronary vessels. The imaging performed by this date, however, had not shown large tumor vessels communicating with the coronary arteries so that these phenomena were supposed to be caused by a multitude of small tumor vessels rather than large communicating vessels that would be amenable to interventional treatment such as embolization.

Assuming that a sufficient reduction of the tumor size would not only palliate the patient’s angina but also reduce the frequency and severity of his ventricular arrhythmia, and given both the surgical risk of an aggressive procedure (Euroscore 14.02 for a combined procedure including CABG) and his palliative situation and limited life expectancy, he was offered a restrictive surgical approach consisting of debulking of the metastatic tumor with an option for subsequent coronary intervention, should the surgical procedure not achieve sufficient palliation of his symptoms.

After median sternotomy and opening of the pericardium, the apex of the heart appeared plump and enlarged with an even surface that showed no visible tumor growth. The large metastasis seen on MRI and CT was palpable on and infiltrating the apex. Cardiopulmonary bypass (CPB) was established in customary fashion because exposition of the apex was not possible without provoking severe arrhythmia and hemodynamic instability. Felt strips were placed along the edges of the palpable tumor mass using 12 pledgeted 4.0 prolene sutures (Fig. 1c) that were snugged down after application of French glue. Without entering the ventricular cavities, a portion of the tumor measuring 27 × 27 × 12 mm was removed in toto (Fig. 1d). Additionally, an approximately equal quantity of fragmented tumor tissue was removed so that only those sections of the tumor that had grown deep into the ventricular walls and septum were left. This resulted in considerable bleeding from a multitude of small vessels within the tumor. After careful hemostasis, the edges of the crater that was left within the area delimited by the felt strips were approximated by tying down two pledgeted 2.0 prolene sutures that were spanned across the crater through two of the felt strips. Following this, the remaining 4.0 sutures were tied down, and the defect was finally closed by a mattress suture followed by an over-and-over suture. The patient remained hemodynamically stable throughout the procedure, and weaning from CPB, decannulation, and sternal closure were performed in customary fashion.

Histopathologic examination of the removed tissue showed a mostly solid tumor consisting of medium-sized to large cells with a wide eosinophilic cytoplasm surrounding enlarged vesicular nuclei with prominent eosinophilic nucleoli. Further findings consisted of atypical mitotic figures and local invasion of blood vessels. Immunohistochemical examination yielded a profile that was also compatible with a diagnosis of metastatic HCC (co-expression of Hep-Par1, positive for CK8 at least in some sections, cytoplasmatic positivity for TTF1, negative for CK7).

The patient was extubated 5 h after the procedure and discharged from the ICU on postoperative day 2. He took an uneventful further course and was discharged on postoperative day 9 with his symptoms palliated. One and a half months later, he presented for a follow-up examination, this time complaining not of cardiac symptoms but of headaches. MRI of the head showed no cerebral metastases. MRI of the heart showed a mass of felt strips and organized hematoma on the apex as well as the change in configuration of the apical region brought about by the surgical procedure. This made it difficult to assess the size of the tumor which appeared, however, to have regained much of its previous size. Nevertheless, the patient survived five and a half months after the procedure with his cardiac symptoms alleviated.

The patient provided written approval of the publication of his case including any pertinent images for scientific purposes.

## Discussion and conclusions

With ratios from 30:1 to 100:1 reported from different studies [2–6], metastatic tumors to the heart are far more frequent than primary cardiac neoplasms. While liver cancer and HCC, in particular, were found to be among the most frequent cancers and causes of cancer death worldwide, cancers of the liver and biliary tract were found to be among the less frequent primary tumors causing cardiac metastases [7, 8]. From different series, cardiac metastases were reported to have been present in 2–4% of HCC patients [3, 9, 10].

Most frequently, cardiac metastases are caused by the HCC invading the vascular system so that the majority of its cardiac metastases are continuous with the intrahepatic HCC and located within the right atrium and right ventricle [9]. Isolated cardiac metastases, in contrast, are rare. When they occur, they may be located within the cardiac cavities, or infiltrate the myocardium, or both. In the case of infiltrative growth, they were reported to be most frequently encountered in the left ventricular free wall and septum [3]. In our case, the location of the tumor was extremely rare in that it infiltrated the walls of both ventricles as well as the interventricular septum.

For cardiac metastatic tumors irrespective of the primary tumor, the symptomology was suggested to be mostly determined by their location rather than the type of primary tumor, with no strong correlation between the extent of cardiac involvement and clinical manifestations. Additionally, a considerable share of cardiac metastases were reported to remain clinically silent and/or to be diagnosed only upon autopsy [2].

In the case of metastases of HCC spreading into the right atrium through the inferior vena cava, symptoms of heart failure caused by increasing occlusion of the cardiac cavities must be expected. In fact, the most common symptoms of cardiac metastases of HCC reported from a retrospective analysis of 48 patients with metastases in the cardiac cavities included bilateral lower leg edema and exertional dyspnea [11]. According to other reports, patients also presented with venous dilatation in the abdominal wall and ascites [12, 13], tachycardia or tachyarrhythmia [3], or chest pain [14].

Our patient, in whom the metastatic tumor did not occlude the cardiac cavities but infiltrated the myocardium, complained from angina and arrhythmia while hemodynamic compromise and symptoms of heart failure were absent. Additionally, our case was highly specific in that CAD was simultaneously present, and it was difficult to determine whether the patient’s symptoms were attributable to CAD or to the metastatic tumor. Evaluation of the patient’s coronary angiography, however, suggested that the metastatic tumor rather than the unaddressed coronary lesions were responsible for the patient’s debilitating symptoms. After all, his RCA lesion had already been addressed, occlusion of the LAD was chronic with good collateralization, and the lesions in the RCX were minor. Considering this, he was accepted for palliative surgery.

Surgical removal of HCC metastases to the heart from the right atrium [15–17] or the right ventricle [18–20] was described previously, with surgery usually performed to palliate symptoms of congestive heart failure or relieve hemodynamic compromise due to right outflow tract obstruction. Additionally, survival benefits were reported for patients with HCC and tumor thrombus in the inferior vena cava and right atrium [5]. While it may be gathered from these reports that carefully selected patients may indeed benefit from palliative surgery, literature provides little guidance on the surgical treatment of metastatic HCC to the heart. There is wide agreement, however, that the use of aggressive surgery to remove tumors metastatic to the heart should be restrictive and reserved to cases where the life expectancy is sufficiently long to justify surgery as the most promising option to palliate symptoms [21, 22].

Even though coronary 3 vessel disease was simultaneously present in our patient, our surgical strategy was limited to palliative debulking of the tumor. The growth of the tumor on and infiltrating the ventricular walls without presence of intracavitary tumor masses enabled us to limit the surgical procedure to debulking without aortic cross clamping and entering of the cardiac cavities. CPB was required, even though an off-pump procedure would have been preferable, because of arrhythmia and hemodynamic instability caused by our attempts to expose the apex. This focus on reduced invasiveness and avoidance of CPB and cross-clamping contrasts with other reports on palliative surgery for isolated metastatic HCC where CPB with cardioplegic arrest [23, 24] was used or surgery was performed in profound hypothermic circulatory arrest [12, 25].

The decision to restrict the surgical intervention to debulking without concomitant CABG due to the patient’s high risk profile and palliative situation was retrospectively proven correct by the fact that debulking alone sufficed to relieve the patient’s symptoms even without a subsequent coronary intervention. This outcome additionally suggests that a relevant share of both angina and arrhythmia had in fact been attributable to the metastatic tumor rather than the unaddressed coronary lesions.

The patient, finding his symptoms palliated and his quality of life improved, benefited from the surgical intervention even though it did not change the dismal prognosis of his cancer and did not address the CAD. The conclusion we draw from our case is that palliative surgery for metastases to the heart may benefit patients, provided that surgery is only offered to palliate symptoms rather than performing extensive and high-risk cardiac procedures concomitantly addressing CAD or valve disease. The findings from our case are relevant because combinations of metastatic HCC of the heart and CAD or valve disease may well occur and raise questions with regard to the appropriate treatment strategy. With interventional treatment options available not only for CAD but also for valve disease, it may be advisable to limit cardiac surgery to addressing the metastatic tumor with an option for subsequent interventional treatment of cardiac disease.

## Abbreviations

CABG: Coronary artery bypass grafting
CAD: Coronary artery disease
CPB: Cardiopulmonary bypass
CT: Computed tomography
HCC: Hepatocellular carcinoma
LAD: Left anterior descending artery
MRI: Magnetic resonance imaging
NSTEMI: Non-ST-elevation myocardial infarction
RCA: Right coronary artery
RCX: Circumflex artery
